# Drug Interactions With Tamoxifen and Treatment Effectiveness in Premenopausal Breast Cancer Patients: A Bayesian Joint Modeling Approach

**DOI:** 10.1002/pds.70157

**Published:** 2025-05-14

**Authors:** Kirsten M. Woolpert, Deirdre P. Cronin‐Fenton, Per Damkier, Anders Kjærsgaard, Stephen Hamilton‐Dutoit, Bent Ejlertsen, Richard F. MacLehose, Peer Christiansen, Rebecca A. Silliman, Timothy L. Lash, Thomas P. Ahern, Lindsay J. Collin

**Affiliations:** ^1^ Department of Clinical Epidemiology Aarhus University and Aarhus University Hospital Aarhus Denmark; ^2^ Department of Clinical Medicine Aarhus University and Aarhus University Hospital Aarhus Denmark; ^3^ Department of Clinical Pharmacology Odense University Hospital Odense Denmark; ^4^ Department of Clinical Research University of Southern Denmark Odense Denmark; ^5^ Department of Pathology Aarhus University Hospital Aarhus Denmark; ^6^ Danish Breast Cancer Group, Department of Oncology Rigshospitalet Copenhagen Denmark; ^7^ Department of Clinical Medicine University of Copenhagen Copenhagen Denmark; ^8^ Division of Epidemiology & Community Health University of Minnesota School of Public Health Minneapolis Minnesota USA; ^9^ Department of Plastic and Breast Surgery Aarhus University Hospital Aarhus Denmark; ^10^ Section of Geriatrics, Department of Medicine Boston University School of Medicine Boston Massachusetts USA; ^11^ Department of Epidemiology Rollins School of Public Health, Emory University Atlanta Georgia USA; ^12^ Winship Cancer Institute, Emory University Atlanta Georgia USA; ^13^ Department of Surgery The Robert Larner, M.D., College of Medicine at the University of Vermont Burlington Vermont USA; ^14^ Department of Population Health Sciences Huntsman Cancer Institute, University of Utah Salt Lake City Utah USA

**Keywords:** breast neoplasms, drug interactions, joint modeling, pharmacoepidemiology, tamoxifen

## Abstract

**Purpose:**

Tamoxifen is guideline treatment for premenopausal women with estrogen receptor‐positive (ER+) breast cancer. Therapeutic efficacy relies partly on tamoxifen biotransformation by CYP2D6, CYP2C19, and CYP3A4 enzymes. We conducted a cohort study to evaluate whether concomitant prescription of drugs that inhibit these enzymes impacted breast cancer recurrence.

**Methods:**

We enrolled 4493 premenopausal women with stage I–III ER+ breast cancer (2002–2011) treated with tamoxifen. We defined time‐varying CYP‐inhibiting drug exposures as the proportion of overlapping days during the tamoxifen treatment period. We estimated associations of concomitant medication use with recurrence using: (1) Bayesian joint modeling (hazard ratio [HR] and 95% credible intervals [95% CrI]), (2) traditional Cox regression (HR and 95% confidence intervals [95% CI]).

**Results:**

During tamoxifen therapy, 13% of the cohort used strong CYP2D6 inhibitors, 31% weak CYP2D6 inhibitors, 37% CYP2C19 inhibitors, and 12% CYP3A4/5 inhibitors. Bayesian joint models showed that women with ≥ 50% overlap between tamoxifen and CYP2D6 inhibitors had increased recurrence risk compared with 0% overlap (HR: 1.24, 95% CrI: 0.96, 1.58). No recurrence association was seen for CYP2C19 inhibitors (≥ 50% vs. 0%, HR = 1.0, 95% CrI: 0.69, 1.40), but traditional Cox models yielded positive associations for CYP2C19 overlap (≥ 50% vs. 0%, HR = 1.45, 95% CI: 1.07, 1.96). With Bayesian joint models, we observed no association between ≥ 50% versus 0% overlap with CYP3A4/5 inhibitors (HR: 0.84, 95% CrI: 0.32, 1.93).

**Conclusions:**

With Bayesian joint modeling, we saw a slight increase in recurrence among CYP2D6‐inhibitor users, but no increase among CYP2C19‐ or CYP3A4‐inhibitor users. Results from Cox regression models were less plausible.

**Summary**

- The study analyzed 4493 premenopausal women with stage I–III estrogen receptor‐positive breast cancer, examining the impact of concomitant use of tamoxifen and CYP2D6‐, CYP2C19‐, and CYP3A4‐inhibiting drugs on recurrence.
- The time‐varying hazard of recurrence, paired with a tendency for higher comedication use in the early period after breast cancer diagnosis, led to improbable results under Cox proportional hazards modeling.
- Bayesian joint modeling offers a potential solution by simultaneously incorporating the longitudinal measurement of the enzyme‐inhibiting prescriptions with the time‐to‐event outcome model for recurrence.
- Concomitant use of CYP2D6 inhibitors and tamoxifen was associated with a slight increase in breast cancer recurrence hazard compared with tamoxifen‐only users.
- Use of CYP2C19‐ and CYP3A4‐inhibiting medications during tamoxifen treatment was not associated with breast cancer recurrence.

## 1 Introduction

Tamoxifen approximately halves the risk of recurrence among women diagnosed with estrogen receptor (ER)‐positive (ER+) breast cancer [1]. At least 5 years of tamoxifen is the guideline adjuvant endocrine treatment for ER+ premenopausal patients, and for ER+ postmenopausal patients for whom aromatase inhibitor therapy is contraindicated [2, 3]. Unfortunately, patients prescribed adjuvant tamoxifen sometimes relapse due to either *de novo* or acquired drug resistance [4]. Tamoxifen is biotransformed to its most potent metabolites by cytochrome P450 (CYP) enzymes. Activities of key enzymes in tamoxifen activation (CYP2D6, CYP2C19, and CYP3A4/5) can be modified by functional polymorphisms in their encoding genes and by co‐administration of medications that are competitive or inhibitory substrates [5, 6].

Several studies have evaluated how CYP2D6 inhibition affects tamoxifen metabolite levels and breast cancer outcomes in treated women [5, 6, 7, 8, 9, 10, 11]. Most focused on selective serotonin reuptake inhibitor (SSRI) antidepressants, which are frequently co‐prescribed with tamoxifen and vary in the degree to which they compete for or inhibit CYP2D6 activity [7, 10, 12, 13]. Administration of the strong CYP2D6 inhibitor paroxetine reduced plasma endoxifen concentrations in tamoxifen‐treated women, and the magnitude of reduction depended on whether genetic impairment of CYP2D6 activity was also present [5, 6]. While pharmacokinetic studies demonstrate this interaction at the molecular level, studies evaluating clinical outcomes have found mixed associations between CYP2D6‐inhibiting co‐medications and rates of recurrence or mortality among tamoxifen‐treated women [7, 8, 9, 10]. For instance, Haque et al. reported that women taking paroxetine for more than 75% of the first year of tamoxifen therapy had 1.20 times the hazard of recurrence (95% CI 0.97, 1.49) compared with nonusers; however, this association appeared to decrease as a function of tamoxifen therapy duration [9].

**FIGURE 1:**
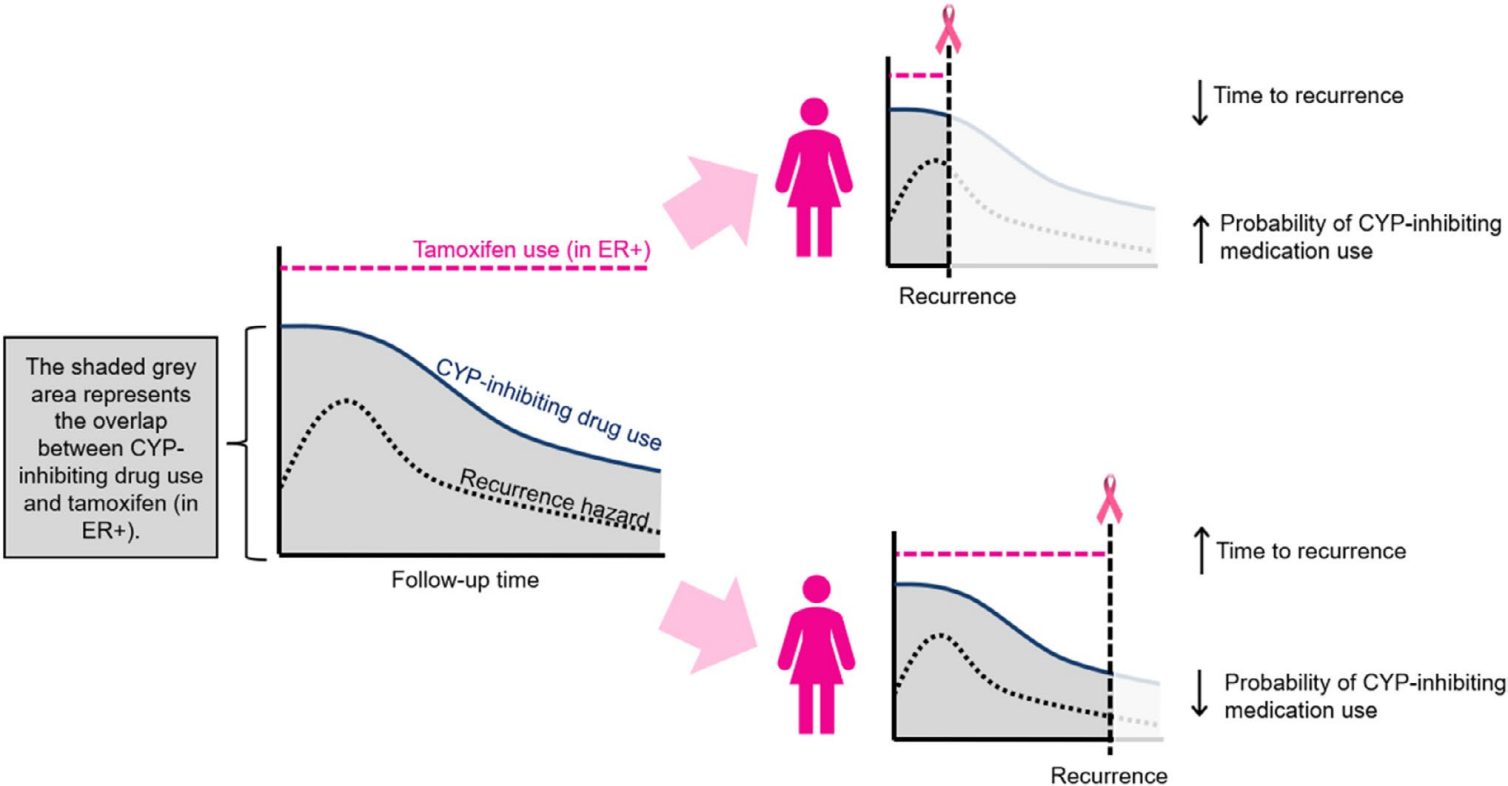
Illustration of the potential issues with using conventional analyses to investigate the relationship between concomitant cytochrome P450 (CYP) inhibitor use and breast cancer recurrence risk. In the top scenario, early recurrence is associated with a higher likelihood of CYP inhibitor use, regardless of recurrence status. In the bottom scenario, late recurrence occurs when CYP inhibitor use is less common. Failing to account for the interplay between time, recurrence risk, and CYP inhibitor use may lead to misleading results. CYP, cytochrome P450; ER, estrogen receptor.

Measuring the impact of concomitant medication use on tamoxifen efficacy is complicated by the tendency to observe higher proportions of overlap during the early phase of therapy when there is also a high initial hazard of recurrence. The proportion of overlap is therefore a function of follow‐up time. This relationship can give the appearance of a decreased response to tamoxifen due to high overlap of co‐medication use when compared with low overlap or nonuse. These latter categories are more likely to be observed over longer follow‐up, which includes a larger share of person‐time after the early peak in recurrence hazard (Figure 1). Joint modeling may offer a solution to this problem by simultaneously incorporating the longitudinal measurement of CYP‐inhibiting prescriptions with the time‐to‐event model for recurrence [14, 15]. Joint modeling was developed as an analytic approach to a continuous exposure with repeated measurements and a survival outcome and has expanded to other longitudinal exposure settings [16, 17, 18]. Additional benefits of this approach are that it accounts for a survival event that terminates the measurement process for the exposure, and for an underlying measurement process that affects the hazard for recurrence, reducing bias in the estimates of the overall exposure effect [14, 16].

We measured the association between pharmaceutical inhibition of CYP2D6, CYP2C19, and CYP3A4 and recurrence in a large cohort of tamoxifen‐treated Danish premenopausal breast cancer patients using a Bayesian joint modeling approach and compared this with traditional Cox regression models.

## 2 Methods

### 2.1 Study Population

This study used the Predictors of Breast Cancer Recurrence (ProBe CaRe) cohort, which consists of 5959 premenopausal women diagnosed with stage I–III breast cancer between 2002 and 2011 who were registered in the Danish Breast Cancer Group (DBCG) clinical database [19, 20]. Demographic, tumor, and treatment data were collected at baseline, and enrollees were actively followed for up to 10 years to detect recurrence. Enrollment was limited to: (1) women with ER+ tumors who were treated with tamoxifen (ER+/T+), and (2) women with ER− tumors who were not treated with tamoxifen (ER−/T−). The latter served as a negative control group to identify residual confounding, as we did not expect the drugs to influence recurrence other than through modification of tamoxifen effectiveness.

### 2.2 Analytic Variables

#### 2.2.1 Drug Exposures

The Danish National Prescription Registry (DNPR) has collected data from all prescription drug transactions at Danish pharmacies since 1995 [21]. We defined categories of exposure to at least one inhibitor of CYP2D6, CYP2C19, and CYP3A4. CYP2D6 inhibitors were separated into categories of strong and weak inhibition, as defined by the US Food and Drug Administration [22]. Table 1 shows the constituent drugs, their ATC codes, and their proportional contribution to each exposure category. In the ER+/T+ stratum, time‐varying CYP‐inhibiting medication exposures were defined based on the proportion of cumulative overlap with tamoxifen treatment (0%, > 0%–50%, ≥ 50%) within each 3‐month interval (i.e., 0–3 months, 3–6 months, etc.) from the start of tamoxifen therapy. These exposure categories were time‐varying, meaning that individuals could transition between categories over the discrete follow‐up periods as their cumulative overlap changed. The same approach was used in the ER−/T− stratum; however, in this context, ‘overlap’ refers to the time a patient was taking a CYP‐inhibiting medication relative to the period when they would have been on tamoxifen had they been ER+, rather than actual concurrent use of both medications. We also characterized exposure to simvastatin (ATC code C10AA01) from DNPR because of its potential to confound associations between other drugs and recurrence [23]. We assumed that each prescription was for a 90‐day supply and that the patient was adherent to both medications.

**TABLE 1:** Medications comprising the CYP2D6, CYP2C19, and CYP3A4/5 inhibitor exposure categories.

Exposure category	Generic medication name	ATC code(s)	Contribution to exposure category, number of prescriptions (%)—ER+	Contribution to exposure category, number of prescriptions (%)—ER−
CYP2D6 inhibitors, strong	Fluoxetine	N06AB03, N06CA03	2341 (23)	661 (24)
	Paroxetine	N06AB05	3042 (30)	666 (24)
	Buproprion	N06AX12, A08AA62	249 (2.4)	57 (2.0)
	Quinidine	C01BA01, C01BA51, C01BA71	0 (0)	0 (0)
	Terbinafine	D01AE15, D01BA02	1757 (17)	480 (17)
	Levomepromazine	N05AA02	1189 (12)	427 (15)
	Duloxetine	N06AX21	1611 (16)	518 (18)
	Moclobemide	N06AG02	74 (0.7)	0 (0)
CYP2D6 inhibitors, weak	Mirtazapine	N06AX11	3775 (9.2)	939 (8.6)
	Amitriptyline	N06AA09, N06CA01	2350 (5.8)	593 (5.4)
	Propranolol	C07AA05, C07FX01, C07BA05	2553 (6.3)	785 (7.2)
	Pindolol	C07AA03, C07CA03	^a^ (0.1)	^a^ (0.1)
	Zuclopenthixol	N05AF05	883 (2.2)	29 (0.2)
	Amiodarone	C01BD01	43 (0.1)	29 (0.2)
	Celecoxib	L01XX33, M01AH01	539 (1.3)	190 (1.7)
	Cimetidine	A02BA01, A02BA51	1028 (2.5)	284 (2.6)
	Venlafaxine	N06AX16	5893 (14)	1293 (12)
	Diltiazem	C05AE03, C08DB01	312 (0.8)	36 (0.3)
	Diphenhydramine	D04AA32, R06AA02, D04AA33, R06AA52	40 (0.1)	9 (0.1)
	Citalopram	N06AB04	11 476 (28)	2828 (26)
	Escitalopram	N06AB10	3162 (7.8)	1143 (10)
	Febuxostat	M04AA03	0 (0)	0 (0)
	Gefitinib	L01XE02	0 (0)	0 (0)
	Hydralazine	C02DB02, C02LG02	0 (0)	0 (0)

	Hydroxychloroquine	P01BA02	174 (0.4)	72 (0.1)
	Imatinib	L01XE01	0 (0)	0 (0)
	Methadone	N07BC02, N02AC52	889 (2.2)	321 (2.9)
	Propafenone	C01BC03	^a^ (0.1)	^a^ (0.1)
	Ranitidine	A02BA02, A02BA07	596 (1.5)	209 (1.9)
	Ritonavir	J05AX66, J05AR10, J05AX67, J05AE03	0 (0)	0 (0)
	Sertraline	N06AB06	3707 (9.1)	1045 (9.6)
	Verapamil	C09BB10, C08DA01, C08DA51	376 (0.9)	160 (1.5)
Metoclopramide	A03FA01	2937 (7.2)	866 (7.9)
CYP2C19 inhibitors	Fluconazole	J01RA07, D01AC15, J02AC01	8486 (53)	2129 (53)
	Fluvoxamine	N06AB08	116 (0.7)	0 (0)
	Omeprazole	A02BC01, A02BD05, A02BD01	4784 (30)	1046 (26)
	Esomeprazole	A02BC05, A02BD06, M01AE52	2774 (17)	814 (20)
CYP3A4/5 inhibitors	Ketoconazole	D01AC08, G01AF11, J02AB02	1949 (49)	637 (56)
	Itraconazole	J02AC02	1096 (27)	256 (22)
	Posaconazole	J02AC04	0 (0)	0 (0)
	Voriconazole	J02AC03	^a^ (< 1)	^a^ (< 1)
	Clarithromycin	J01FA09, A02BD06, A02BD07, A02BD09, A02BD05, A02BD04, A02BD11	958 (24)	249 (22)
	Ritonavir	J05AX66, J05AR10, J05AX67, J05AE03	0 (0)	0 (0)

	Nelfinavir	J05AE04	0 (0)	0 (0)
	Saquinavir	J05AE01	0 (0)	0 (0)
	Telaprevir	J05AE11	0 (0)	0 (0)
	Indinavir	J05AE02	0 (0)	0 (0)
Cobicistat	J05AR15, V03AX03, J05AR14, J05AR18, J05AR09	0 (0)	0 (0)

^a^ Number not shown in accordance with Danish data privacy laws (prescriptions redeemed by less than 5 patients).

In sensitivity analyses, we also defined a binary (yes/no) comedication exposure in each 3‐month interval, and changed our assumption of a 90‐day supply to a 30‐day supply, as described in the Supporting Information S1. Drug exposures were lagged by 1 year to guard against possible influence of incipient recurrences on drug prescribing patterns.

#### 2.2.2 Recurrence

We used the recurrence definition adopted by DBCG, which includes any type of invasive breast cancer (local or contralateral) or distant metastasis diagnosed after a patient completes primary and adjuvant therapies [20].

#### 2.2.3 Covariates

We collected demographic information (age at diagnosis), tumor characteristics (tumor size and lymph node status used to derive UICC pathologic stage, histological grade, and human epidermal growth factor receptor‐2 [HER2] expression status), and treatment data (type of primary surgery [mastectomy or breast‐conserving surgery], chemotherapy, and radiation therapy) from the DBCG. Unambiguous linkage of data from the DBCG to other Danish registries was accomplished using subjects' civil personal registration (CPR) numbers—unique identifiers assigned to all residents of Denmark upon birth or immigration [24, 25]. Prevalent comorbidities registered in the National Patient Registry were used to derive the Charlson Comorbidity Index (CCI) [26, 27, 28].

#### 2.2.4 Statistical Analysis

We tabulated the frequency and proportion of subjects according to demographic and clinical characteristics by ER/T group. Person‐time at risk for breast cancer recurrence was tallied from 1.5 years after the date of breast cancer surgery until the first date of recurrence diagnosis, new primary cancer diagnosis, death, emigration, 10 years after diagnosis, or the end of available follow‐up on August 31, 2019. The start date of 1.5 years after the date of breast cancer surgery was chosen because a recurrence in that period would likely not be related to tamoxifen resistance. Though this definition may represent the most clinically relevant period for ER+ patients, it also may exclude person‐time with the strongest link between increased co‐medication overlap with tamoxifen and the highest recurrence hazard. Thus, in a sensitivity analysis, we also started follow‐up 6 months after diagnosis. This period was chosen because ER+ women, on average, will initiate tamoxifen at this time.

We used a Bayesian joint modeling approach to simultaneously model the association between longitudinal use of concomitant drugs and breast cancer recurrence [14, 29]. The joint model consists of two components, a generalized linear mixed effects model and a time‐to‐event model. For each drug class, we fit a longitudinal ordinal logistic model with a random effects term for the association between time and proportion overlap categorization. We used Cox proportional hazards regression for the time‐to‐event models. In the joint model, these two submodels are linked through a shared parameter that incorporates the longitudinal exposure trajectory and the outcome hazard through a cumulative effect association structure [14, 30].

We performed these analyses using the “JMbayes2” R package, with noninformative normal priors and a Markov chain Monte Carlo (MCMC) algorithm to sample from the posterior distributions [31]. We report the median and 95% credible interval (CrI) from the posterior distributions. Additional information on our approach is in the Supporting Information S1.

As a comparison, we also fit Cox proportional hazards regression models to estimate associations between time‐varying CYP‐inhibiting medication exposures and breast cancer recurrence. Medication exposures were fit in the model in the same way described for the Bayesian joint models above. Associations are reported as hazard ratios (HRs) with accompanying 95% confidence intervals (CIs).

In both approaches, we report multivariable models adjusting the associations for patient age at diagnosis, cancer stage, surgery type, chemotherapy receipt, radiotherapy receipt, CCI, and time‐varying simvastatin use within joint strata of ER expression and tamoxifen treatment (i.e., ER+/T+ and ER−/T−). Women in the ER+/T+ group who became postmenopausal and switched from tamoxifen to an AI were censored at the time of the switch. Analyses were carried out with SAS v9.4 (Cary, North Carolina) and R v3.6 (R Foundation, Vienna, Austria).

## 3 Results

### 3.1 Cohort Characteristics

The ProBe CaRe cohort enrolled 5959 premenopausal breast cancer survivors: 4600 in the ER+/T+ stratum and 1359 in the ER−/T− stratum. In our main analysis, 262 women (107 ER+/T+, 155 ER−/T−) were excluded because they had less than 1.5 years of follow‐up, for a final cohort of 4493 ER+/T+ and 1204 ER−/T− patients. Among both ER strata, the most prescribed inhibitors were citalopram for CYP2D6, fluconazole for CYP2C19, and ketoconazole for CYP3A4/5 (Table 1). Among ER+/T+ women, 583 (13%) received a strong CYP2D6 inhibitor, 1436 (31%) a weak CYP2D6 inhibitor, 1707 (37%) a CYP2C19 inhibitor, and 532 (12%) a CYP3A4/5 inhibitor during tamoxifen therapy (Table 2). Overlap between inhibitor exposures was common (Table 2). Distributions of age at diagnosis, tumor stage, histological grade, HER2 status, and receipt of chemotherapy were similar across exposure categories.

**TABLE 2:** Baseline characteristics of the cohort according to use of cytochrome P450‐inhibiting medications.^a^

	Users of strong inhibitors of CYP2D6, *n* (%)	Users of weak inhibitors of CYP2D6, *n* (%)	Users of inhibitors of CYP2C19, *n* (%)	Users of inhibitors of CYP3A4/5, *n* (%)	Nonusers of any CYP inhibitor, *n* (%)
Total^a^	583 (13)	1436 (32)	1707 (38)	532 (12)	1837 (41)
Age at diagnosis					
< 40	88 (15)	183 (13)	291 (17)	81 (15)	268 (15)
40–49	345 (59)	899 (63)	1057 (62)	313 (59)	1111 (61)
50–59	150 (26)	354 (25)	359 (21)	138 (26)	458 (25)
UICC stage^b^					
I	164 (28)	378 (26)	473 (28)	143 (27)	454 (25)
II	304 (52)	759 (53)	919 (54)	296 (56)	995 (54)
III	115 (20)	299 (21)	315 (18)	93 (17)	388 (21)
Histological grade					
Low	115 (20)	290 (22)	342 (21)	115 (23)	384 (22)
Moderate	324 (58)	787 (59)	908 (57)	287 (58)	915 (53)
High	121 (22)	260 (19)	346 (22)	95 (19)	413 (24)
Missing/not graded	23	99	111	35	125
HER2 status					
Positive	78 (19)	165 (15)	228 (17)	70 (18)	260 (19)
Negative	343 (82)	922 (85)	1099 (83)	329 (83)	1116 (81)
Missing/not tested	162	349	380	133	461
Surgery type					
Mastectomy	282 (49)	652 (45)	761 (45)	232 (44)	783 (43)
BCS + RT	301 (52)	784 (55)	946 (55)	300 (56)	1054 (57)
Chemotherapy	572 (98)	1395 (97)	1655 (97)	517 (97)	1779 (97)
Other inhibitors					
Strong CYP2D6	—	303 (21)	268 (16)	92 (17)	—
Weak CYP2D6	303 (52)	—	685 (40)	240 (45)	—
CYP2C19	268 (46)	685 (48)	—	288 (54)	—
CYP3A4/5	92 (16)	240 (17)	288 (17)	—	—
Simvastatin	57 (9.8)	130 (9.1)	124 (7.3)	39 (7.3)	71 (3.9)
Charlson Comorbidity Index (CCI)					
0	524 (90)	1276 (89)	1544 (90)	468 (88)	1729 (94)
1+	59 (10)	160 (11)	163 (9.5)	64 (12)	108 (5.9)
Recurrence within 10 years	72 (12)	170 (12)	185 (11)	54 (10)	1729 (14)

*Note:* Premenopausal Danish women diagnosed with stage I, II, or III estrogen receptor positive breast cancer between 2002 and 2011 and treated with adjuvant tamoxifen (*n* = 4493).

^a^ Cohort members may appear in more than one of the columns of users of medications.

^b^ Missing stage was multiply imputed using available participant characteristics. This has been previously described [32].

**FIGURE 2:**
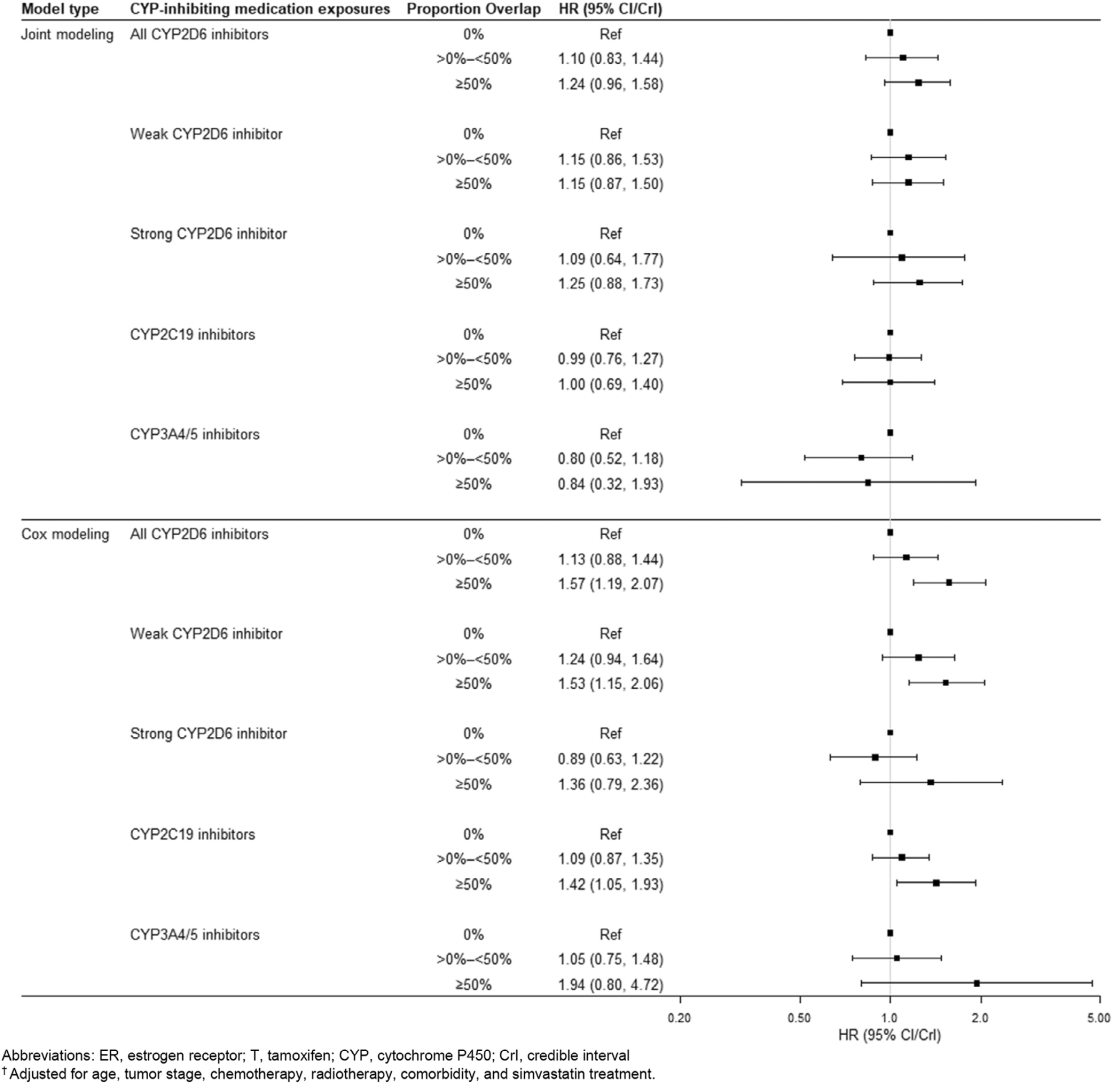
Associations^†^ between proportion overlap of cytochrome P450‐inhibiting co‐medications and breast cancer recurrence, among 4493 estrogen receptor‐positive (ER+) women diagnosed with stage I, II, or III breast cancer between 2002 and 2011 in Denmark. CrI, credible interval; CYP, cytochrome P450; ER, estrogen receptor; T, tamoxifen. ^†^Adjusted for age, tumor stage, chemotherapy, radiotherapy, comorbidity, and simvastatin treatment.

**FIGURE 3:**
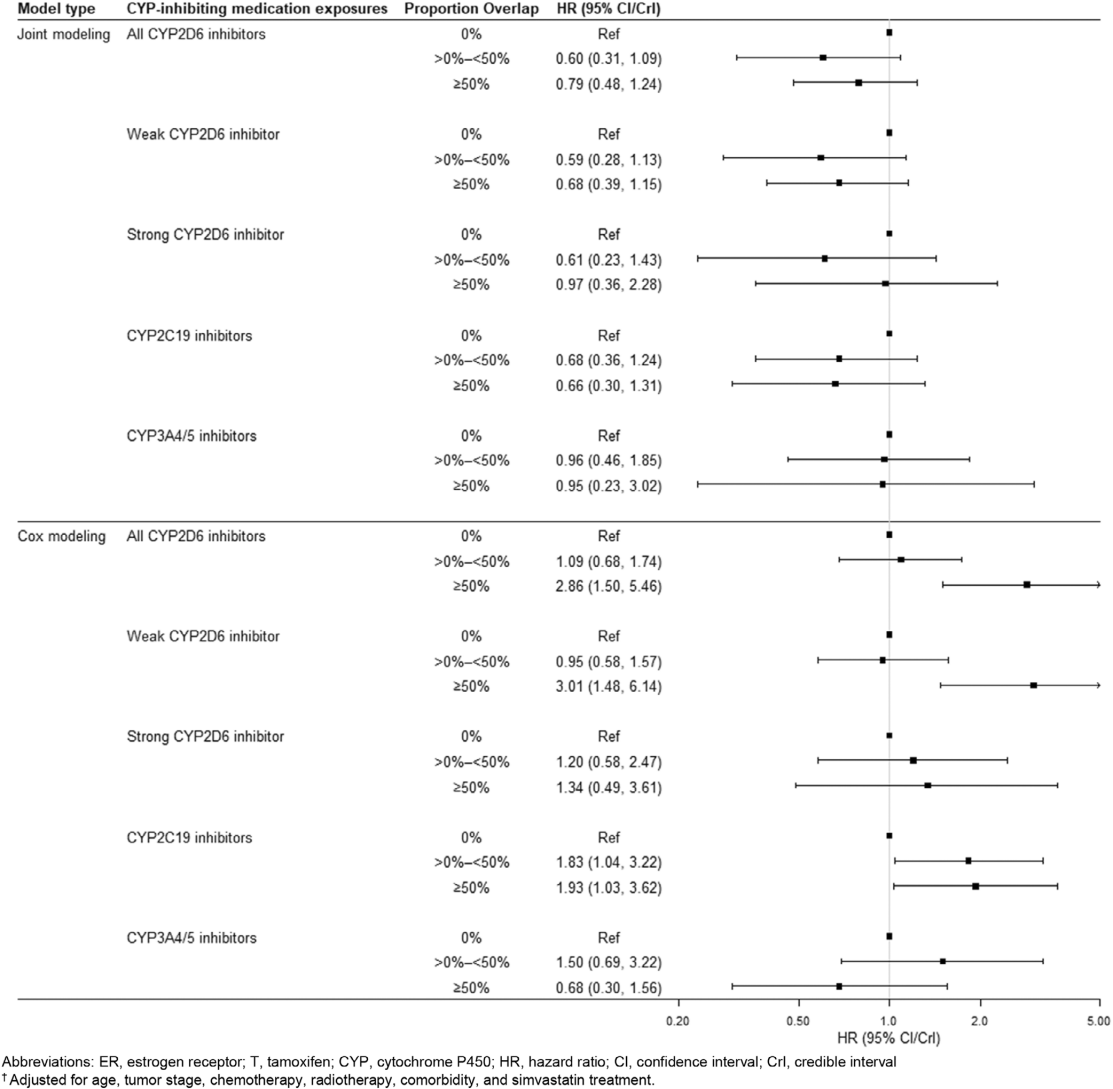
Associations^†^ between proportion overlap of cytochrome P450‐inhibiting co‐medications and breast cancer recurrence, among 1204 estrogen receptor‐negative (ER−) women diagnosed with stage I, II, or III breast cancer between 2002 and 2011 in Denmark. CI, confidence interval; CrI, credible interval; CYP, cytochrome P450; ER, estrogen receptor; HR, hazard ratio; T, tamoxifen. ^†^Adjusted for age, tumor stage, chemotherapy, radiotherapy, comorbidity, and simvastatin treatment.

We report multivariable‐adjusted results among ER+/T+ patients in Figure 2, which can be compared with the same results among ER−/T− (negative control) patients in Figure 3. Crude results were similar to multivariable‐adjusted results and are reported in the Supporting Information S1. Sensitivity analyses using binary CYP‐inhibitor exposure and excluding patients with < 0.5 years of follow‐up are in the appendix; neither differed meaningfully from the main results.

### 3.2 CYP‐Inhibiting Medications and Recurrence

#### 3.2.1 CYP2D6 Inhibitors

When accounting for the longitudinal concomitant medication use with Bayesian joint modeling, we observed an association between any CYP2D6‐inhibiting medication use (versus no CYP2D6‐inhibiting medication use) among the ER+/T+ patients. For example, ER+/T+ patients who were concomitantly prescribed any CYP2D6‐inhibiting medication for more than half of their intended tamoxifen treatment time had a higher recurrence hazard compared with patients not prescribed any CYP2D6 inhibitor (HR = 1.24, 95% CrI: 0.96, 1.58). There was some evidence that this association depended on CYP2D6 inhibitor strength. Compared with nonusers, women with ≥ 50% overlap between tamoxifen and weak CYP2D6 inhibitors had a small but imprecise increase in recurrence rates (HR = 1.15, 95% CrI: 0.57, 1.53). This association strengthened when looking at the same comparison among tamoxifen users with ≥ 50% overlap with strong CYP2D6 inhibitors (HR = 1.25, 95% CrI: 0.88, 1.73). The direction and magnitude of these relationships were similar when compared with conventional models in the ER+/T+ stratum.

In the ER−/T− group using Bayesian joint modeling, we observed null or protective associations between CYP2D6 inhibiting medication use and breast cancer recurrence. This differs from our conventional results using Cox proportional hazards regression, which showed null or harmful associations. For example, when comparing ≥ 50% overlap with any CYP2D6 inhibitor compared with no overlap, we observed an elevated and imprecise HR of 2.86 (95% CI: 1.50, 5.46) from the Cox model in ER−/T− women. This same association was slightly protective, more precise, and overlapped with the null in Bayesian joint modeling analyses (HR: 0.79, 95% CrI: 0.48, 1.24).

#### 3.2.2 CYP2C19 Inhibitors

We observed no association using Bayesian joint models between CYP2C19 inhibiting medication use and breast cancer recurrence among the ER+/T+ patients (i.e., ≥ 50% vs. 0%, HR = 1.0, 95% CrI: 0.69, 1.40). In Cox analyses, CYP2C19 medication use was positively associated with recurrence (i.e., ≥ 50% vs. 0%, HR = 1.45, 95% CI: 1.07, 1.96). For the joint modeling analysis in the ER−/T− group, we observed a protective association comparing ≥ 50% overlap with tamoxifen with no overlap (HR = 0.66, 95% CrI: 0.30, 1.31), which differed from the harmful association observed in the Cox analysis comparing ≥ 50% overlap with tamoxifen with no overlap (HR = 1.93, 95% CI: 1.03, 3.62).

#### 3.2.3 CYP3A4/5 Inhibitors

Compared with no CYP3A4/5‐inhibiting medication use, we observed near‐null and imprecise associations between more than 50% overlap with CYP3A4/5 inhibitors in both the ER+/T+ group (HR: 0.84, 95% CrI: 0.32, 1.93) and the ER−/T− group (HR: 0.95, 95% CrI: 0.23, 3.02) in our Bayesian joint modeling approach. In Cox analyses, these same comparisons yielded a HR of 1.71 (95% CI: 0.70, 4.17) among the ER+/T+ group, and 0.68 (95% CI: 0.30, 1.56) among the ER−/T− group.

## 4 Discussion

This is the first study of the impact of CYP‐inhibiting co‐medications on tamoxifen effectiveness in an exclusively premenopausal cohort. In our joint modeling approach, we observed that tamoxifen‐treated patients with concomitant use of CYP2D6‐inhibiting medications had between a 9% and 25% increased rate of recurrence. For other analyses using the joint modeling approach, nearly all associations between concomitant medication use were null (or near null) among women in both ER/T groups. However, results from the Cox models using a time‐varying exposure of CYP‐inhibiting medication overlap yielded strong associations across nearly all drug classes among users with high overlap in both ER/T strata.

The somewhat increased hazard of breast cancer recurrence among users of CYP2D6 inhibitors concomitantly taking tamoxifen was dependent on both the strength and proportion of overlapping time. Patients taking strong CYP2D6 inhibitors, which included SSRI antidepressants such as citalopram and fluoxetine, for more than half of their intended tamoxifen treatment period had a slightly increased hazard of recurrence. These findings align with prior studies [9, 10], which have prompted recommendations to avoid concomitant medications, especially SSRI antidepressants, that may reduce tamoxifen effectiveness [33, 34]. However, a systematic review showed that there is no consistent negative effect of antidepressant use on breast cancer‐related outcomes, arguing that more rigorously designed studies are needed [35]. Another possible explanation for the inconsistent findings in the literature and the finding of increased risk among CYP2D6‐inhibitor users in this study could relate to tamoxifen adherence. In another study in the ProBe CaRe cohort, we observed that women who were prevalent users of psychoanaleptics at breast cancer diagnosis, including antidepressants, had poorer endocrine therapy adherence [32]. It is then possible that the observed reduction in tamoxifen effectiveness seen among CYP2D6‐inhibiting medication users relates to lower levels of tamoxifen adherence, thus increasing recurrence risk [36].

Use of the other investigated enzyme inhibiting medications—CYP2C19 and CYP3A4—did not show meaningful associations with recurrence. Associations between CYP2C19 use and tamoxifen effectiveness were either null or protective in both ER strata [37]. In our joint modeling approach, CYP3A4/5 inhibitors were not associated with breast cancer recurrence; however, in our Cox modeling approach, CYP3A4/5 inhibitor use was associated with higher breast cancer recurrence risk in ER+ tamoxifen‐treated women vs. nonusers. The medications contributing most to the CYP3A4/5 inhibitor category in our study population are short‐term anti‐fungal medications and are not expected to influence recurrence risk.

Joint models were originally developed in biomarker studies, where the trajectory of the biomarker was incorporated as an exposure—with an acknowledged level of measurement error—in a survival model [15]. We used the receipt of CYP‐inhibiting concomitant medication in the joint model, which accounted for the strong link between a high proportion of tamoxifen overlap in the early follow‐up period where there is also an increased recurrence hazard (Figure 1). The joint models accounted for the recurrence events that terminated the overlap of concomitant medication. The pronounced estimates of association among women with high overlap in the ER−/T− would have indicated an implausible association between the medication and recurrence (independent of reduced tamoxifen efficacy), but the results from the joint modeling approach indicated near‐null or protective associations. The observed protective associations in the ER−/T− stratum are unexpected, but may suggest that breast cancer patients with ER− disease who are sicker from their disease are less likely to receive medication for other conditions (i.e., confounding by indication).

In this study, the joint and Cox models resulted in substantively different estimates of association for many inhibitor classes. Joint models have the advantage of accounting for longitudinal exposure data, which can smooth over random fluctuations in prescription records, such as errors in redemption data. For instance, if a woman is classified as a CYP2D6 inhibitor user for 11 months, a longitudinal model would likely categorize her as a user for the full year. This approach can increase estimate precision by leveraging more data, as seen in many of our joint models. However, misspecification of the time‐exposure relationship in the longitudinal model could lead to less stable estimates compared with the simpler Cox model. Another factor may be reverse causation, though this is less likely in our context, as most medications investigated would not be prescribed for undetected recurrences. Future work should explore why these methods yield such different results.

We used filled prescriptions as a proxy for actual exposure to medications. We assumed that patients were adherent to their prescriptions (for both tamoxifen and for CYP‐inhibiting medications), so the actual overlap between tamoxifen and co‐medications may not be accurately characterized. Despite the relatively large size of our premenopausal cohort, the prevalence of co‐medication exposure was modest, yielding few recurrence cases in some exposure categories. This was particularly true in the ER−/T− stratum. Additionally, channeling bias may be a limitation, as some medications, like SSRIs, are used for sequelae of breast cancer and may reflect sicker patient populations that are at a higher risk for recurrence.

## 5 Conclusion

In this study, we observed a modest indication that co‐medications inhibiting CYP2D6 activity may influence tamoxifen effectiveness in premenopausal women. Inhibition of two other key enzymes, CYP2C19 and CYP3A4/5, did not show clear evidence of an association with recurrence. Bayesian joint modeling provides a potential approach to account for longitudinal patterns of medication use and the nonuniform hazard of recurrence, addressing some of the limitations of previous studies that relied on Cox regression models.

### 5.1 Plain Language Summary

In this study, we analyzed 4493 premenopausal women with early‐stage estrogen‐receptor breast cancer to see how taking tamoxifen with certain enzyme‐inhibiting drugs (CYP2D6, CYP2C19, and CYP3A4 inhibitors) affected the risk of cancer recurrence. Using traditional statistical methods, we found unreliable results, which we hypothesize were due to changes in the recurrence risk over time and more frequent use of other medications soon after diagnosis. To overcome this, we applied a more advanced statistical approach, Bayesian joint modeling, which incorporates both the timing of medication use and cancer recurrence. Our findings showed a slight increase in recurrence risk when CYP2D6 inhibitors were used with tamoxifen, but no increased risk for drugs inhibiting CYP2C19 or CYP3A4.

## Ethics Statement

Approval for this study was granted by the Danish Breast Cancer Group and the Danish Data Protection Agency (Aarhus University number 2016–051‐000001, #458) and adhered to the General Data Protection Regulation. The compilation and analysis of data in this study were conducted within the secure servers of Statistics Denmark, in accordance with Danish privacy laws. In accordance with Danish data privacy laws, these data cannot be shared.

## Conflicts of Interest

L.J.C. reports personal fees from Epidemiologic Research & Methods LLC outside of the submitted work. T.L.L. is a member of the Amgen Methods Advisory Council, for which he receives consulting fees and travel support. This work is outside of the submitted work. B.E. reports outside the submitted work an Advisory Role: Eli Lilly; Research funding: Institutional grants from AstraZeneca, Daiichi Sankyo, Eli Lilly, Gilead, Novartis, Pfizer, and Seagen; and Travel, Accommodations, Expenses: Daiichi Sankyo, MSD, and Pfizer. The Department of Clinical Epidemiology, Aarhus University, receives funding for other studies from the European Medicines Agency and from companies in the form of research grants, unrelated to the current study. All other authors have no disclosures. This project was presented as a poster at the International Conference on Pharmacoepidemiology in Copenhagen, Denmark in August 2022.

## Supporting information


**Data S1.** Supporting Information.
