# Supplementary material for: Drug Interactions With Tamoxifen and Treatment Effectiveness in Premenopausal Breast Cancer Patients: A Bayesian Joint Modeling Approach
Source: Pharmacoepidemiol Drug Saf. 2025 May 14;34(5):e70157. doi: 10.1002/pds.70157 (PMC12076038; doi:10.1002/pds.70157)
Supplement: Supplementary file 1 — Data S1. Supporting Information. [file PDS-34-e70157-s001.docx]

**Online supplement to:** Drug interactions with tamoxifen and treatment effectiveness in premenopausal breast cancer patients: A Bayesian joint modeling approach

**Authors**

Kirsten M. Woolpert (1), Deirdre P. Cronin-Fenton (1), Per Damkier (2,3), Anders Kjærsgaard (1), Stephen Hamilton-Dutoit (4), Bent Ejlertsen (5,6), Richard F. MacLehose (7), Peer Christiansen (5,8), Rebecca A. Silliman (9), Timothy L. Lash (1,10,11), Thomas P. Ahern (12), & Lindsay J. Collin (1,13)

**Table of Contents**

[**Supplemental Methods** 3](#_Toc190423226)

[*Additional methodology for Bayesian joint modeling* 3](#_Toc190423227)

[*Sensitivity analysis – Binary comedication exposure definition* 3](#_Toc190423228)

[*Sensitivity analysis – Tallying person-time at risk for recurrence from 0.5 years after breast cancer surgery* 3](#_Toc190423229)

[*Sensitivity analysis – Length of exposure intervals* 3](#_Toc190423230)

[**Supplemental Results** 4](#_Toc190423231)

[**Supplementary Figure S1**. Flow chart for study inclusion 4](#_Toc190423232)

[**Supplementary Figure S2**. Traceplot for Bayesian joint model estimating the association between proportion overlap of all CYP2D6 inhibiting co-medications and breast cancer recurrence, among estrogen-receptor positive premenopausal breast cancer patients.^†^ 5](#_Toc190423233)

[**Supplementary Figure S3**. Density plots for Bayesian joint model estimating the association between proportion overlap of all CYP2D6 inhibiting co-medications and breast cancer recurrence, among estrogen-receptor positive premenopausal breast cancer patients.^†^ 6](#_Toc190423234)

[**Supplementary Figure S4**. Traceplot for Bayesian joint model estimating the association between proportion overlap of all CYP2D6 inhibiting co-medications and breast cancer recurrence, among estrogen-receptor negative premenopausal breast cancer patients.^†^ 7](#_Toc190423235)

[**Supplementary Figure S5**. Density plots for Bayesian joint model estimating the association between proportion overlap of all CYP2D6 inhibiting co-medications and breast cancer recurrence, among estrogen-negative positive premenopausal breast cancer patients.^†^ 8](#_Toc190423236)

[**Supplementary Table S1**. Crude associations between proportion overlap of cytochrome P450-inhibiting co-medications and breast cancer recurrence 9](#_Toc190423237)

[**Supplementary Table S2**. Crude associations between proportion overlap of cytochrome P450-inhibiting co-medications and breast cancer recurrence 10](#_Toc190423238)

[**Supplementary Table S3**. Sensitivity analysis of associations between binary definition of cytochrome P450-inhibiting co-medications and breast cancer recurrence among 4,493 estrogen receptor-positive (ER+) p 11](#_Toc190423239)

[**Supplementary Table S4**. Sensitivity analysis of associations between binary definition of cytochrome P450-inhibiting co-medications and breast cancer recurrence among 12](#_Toc190423240)

[**Supplementary Table S5**. Associations between proportion overlap of cytochrome P450-inhibiting co-medications and breast cancer recurrence, according to estrogen receptor expression and tamoxifen treatment status. Estimated with Bayesian joint models. Sensitivity analysis with person-time at risk starting six months after diagnosis. 13](#_Toc190423241)

# **Supplemental Methods**

# *Additional methodology for Bayesian joint modeling*

Each Bayesian joint model was run for 3 Markov chain Monte Carlo (MCMC) simulations, 10,000 iterations per chain with 2,000 burn-in iterations. We fit a non-negative cubic spline function (M-splines) on time with three knots placed by default from the splines2 package (forming the basis for ordinary polynomial regression).^1,2^ We investigated MCMC diagnostic plots, including trace and density plots of all parameter estimates.^2–4^ We report selected trace and density plots of the parameter estimate representing the association between proportion overlap of CYP2D6-inhibiting medication and the hazard of breast cancer recurrence (**Supplementary Figures S2-S5**). We also report the crude associations in **Supplementary Tables S1-S2**.

## *Sensitivity analysis – Binary comedication exposure definition*

As sensitivity analysis of our exposure definition, we also defined a time-varying exposure to CYP-inhibiting medications as the receipt of medication (yes/no) in a given 90-day period. As in our main analysis, we assumed that the patient was adherent during this time period, and updated prescription data for the first five years in both ER/T groups (corresponding to the time ER+/T+ patients were concomitantly taking tamoxifen).

With this definition, we compared results using Bayesian joint modeling and conventional Cox proportional hazards modeling. For the Bayesian joint models, we first fit a random effects binomial submodel for the association between time (3-month time intervals after initiation of follow-up time) and binary receipt of medication (yes/no). We then used Cox proportional hazards regression for the time-to-event submodel. Methods for conventional modeling did not differ from the main analysis. Results of this analysis are reported in **Supplementary Tables S3-S4**.

## *Sensitivity analysis – Tallying person-time at risk for recurrence from 0.5 years after breast cancer surgery*

As sensitivity analysis of our study population, we conducted an analysis with the Bayesian joint models starting follow-up for recurrence from 0.5 years after breast cancer diagnosis date. This was to ensure that the exclusion of the earliest follow-up period (where the co-medication overlap and highest recurrence hazard link is strongest) did not meaningfully influence results. These models were run in the same way as described above. Results of this analysis are reported in **Supplementary Table S5**.

## *Sensitivity analysis – Length of exposure intervals*

In our main analysis, we assumed that all medication fills included a 90-day supply, which was the approximate average amount dispensed across all studied medications. For CYP2D6 inhibitors, like selective serotonin reuptake inhibitors (SSRIs), this is consistent with refill patterns for patients. However, CYP2C19 and CYP3A4/5 inhibiting medications largely consist of short-term antibiotic and antifungal medications. We conducted a sensitivity analysis for these two medication groups (CYP2C19 and CYP3A4/5) where we changed this assumption to 30-day fills. The results are reported in **Supplementary Table S6**.

# **Supplemental Results**

# **Supplementary Figure S1**. Flow chart for study inclusion


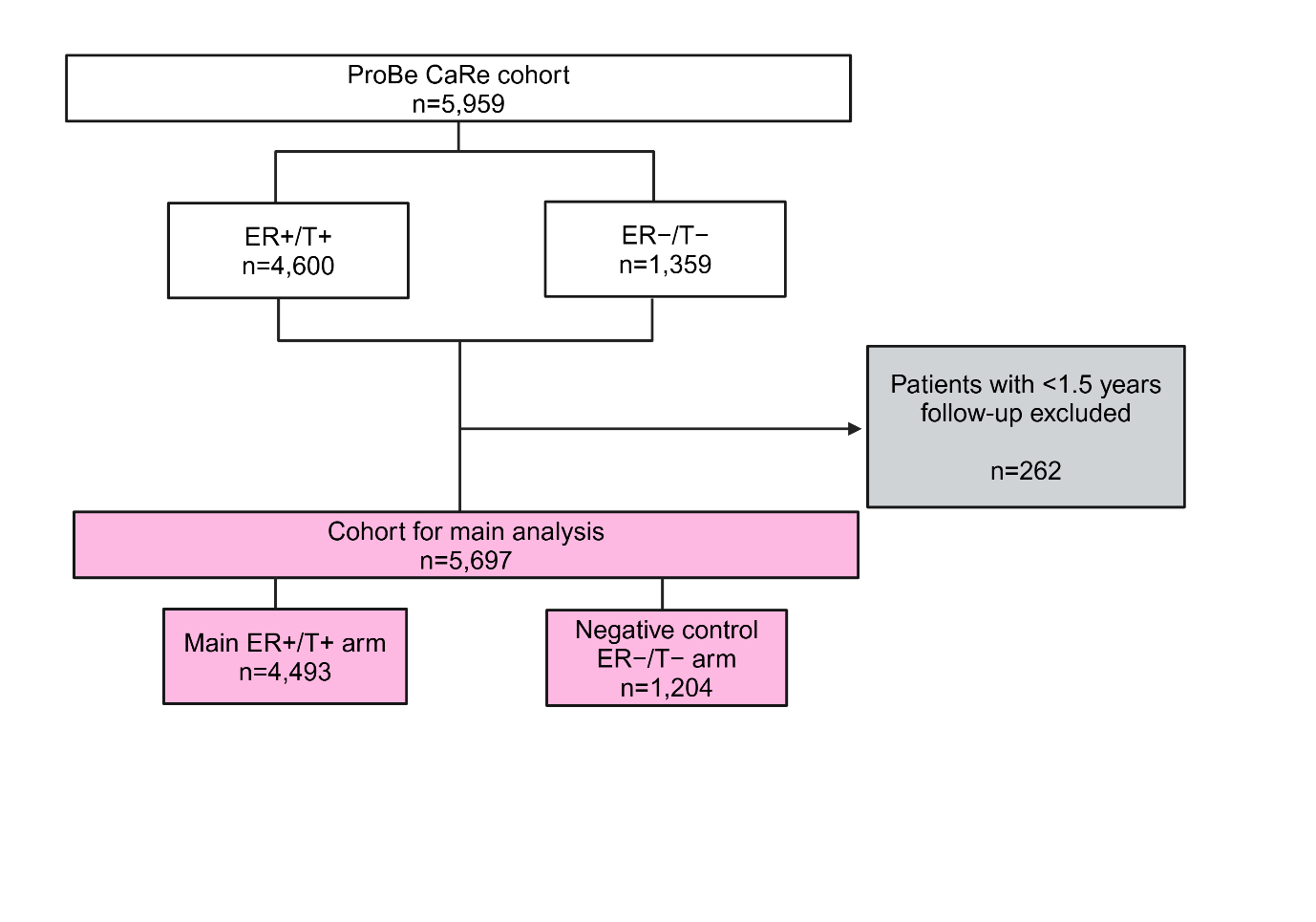


## **Supplementary Figure S2**. Traceplot for Bayesian joint model estimating the association between proportion overlap of all CYP2D6 inhibiting co-medications and breast cancer recurrence, among estrogen-receptor positive premenopausal breast cancer patients.^†^


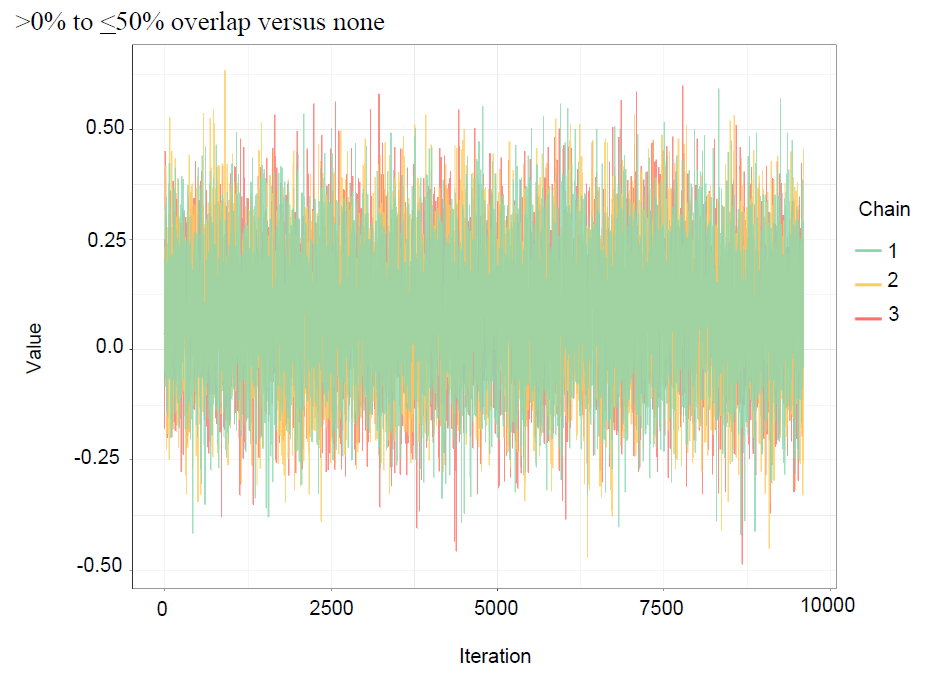


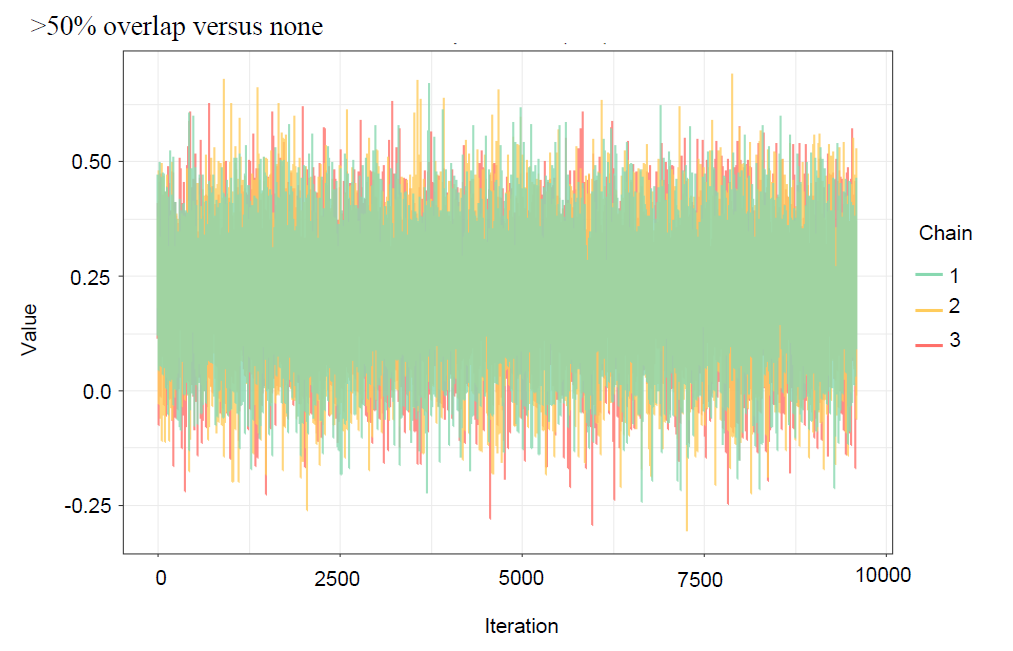


^†^ Adjusted for age, tumor stage, chemotherapy, radiotherapy, comorbidity, and simvastatin treatment.

## **Supplementary Figure S3**. Density plots for Bayesian joint model estimating the association between proportion overlap of all CYP2D6 inhibiting co-medications and breast cancer recurrence, among estrogen-receptor positive premenopausal breast cancer patients.^†^


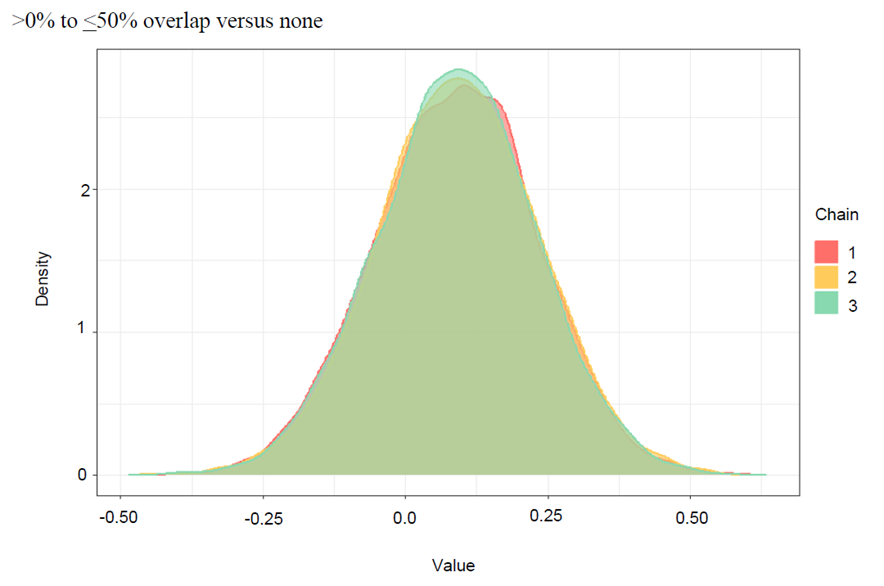


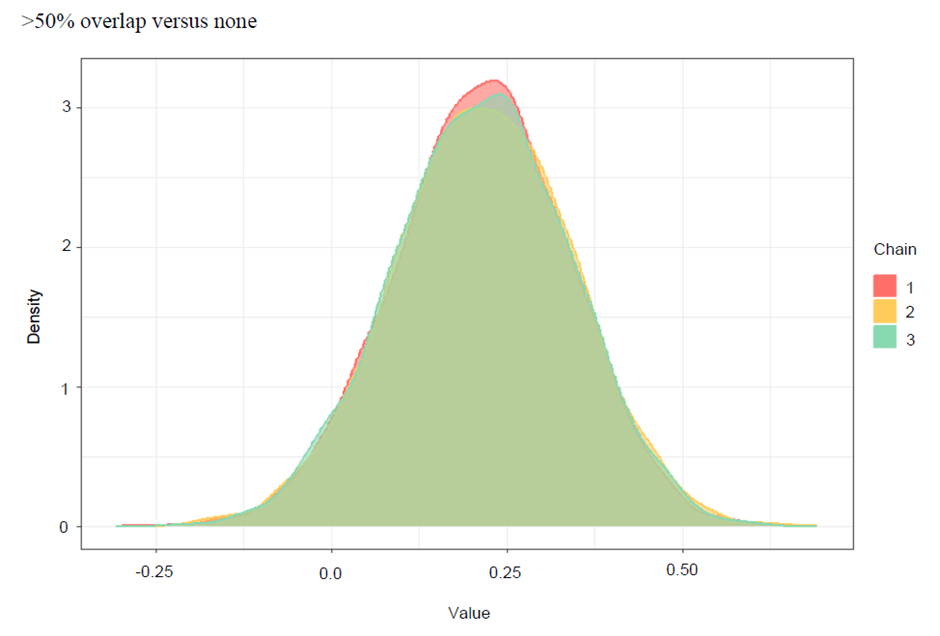


^†^ Adjusted for age, tumor stage, chemotherapy, radiotherapy, comorbidity, and simvastatin treatment.

## **Supplementary Figure S4**. Traceplot for Bayesian joint model estimating the association between proportion overlap of all CYP2D6 inhibiting co-medications and breast cancer recurrence, among estrogen-receptor negative premenopausal breast cancer patients.^†^


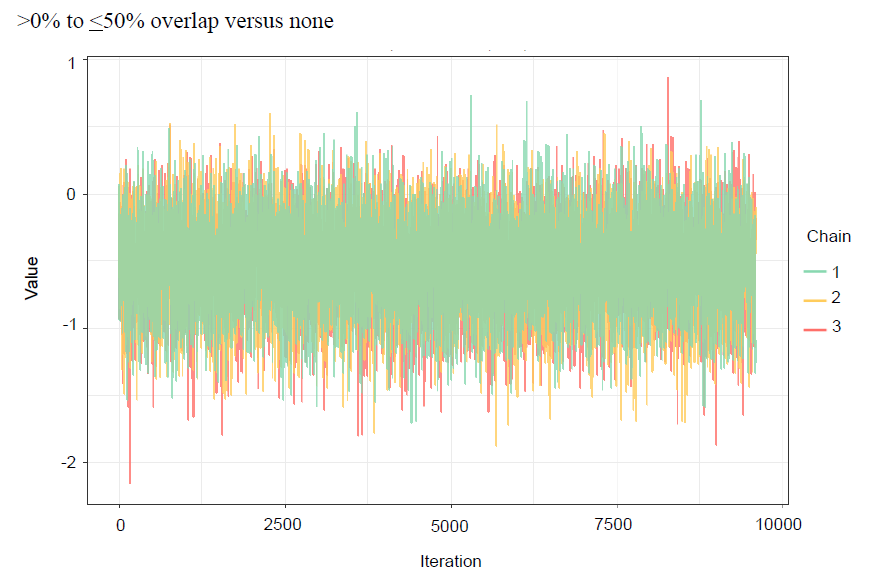


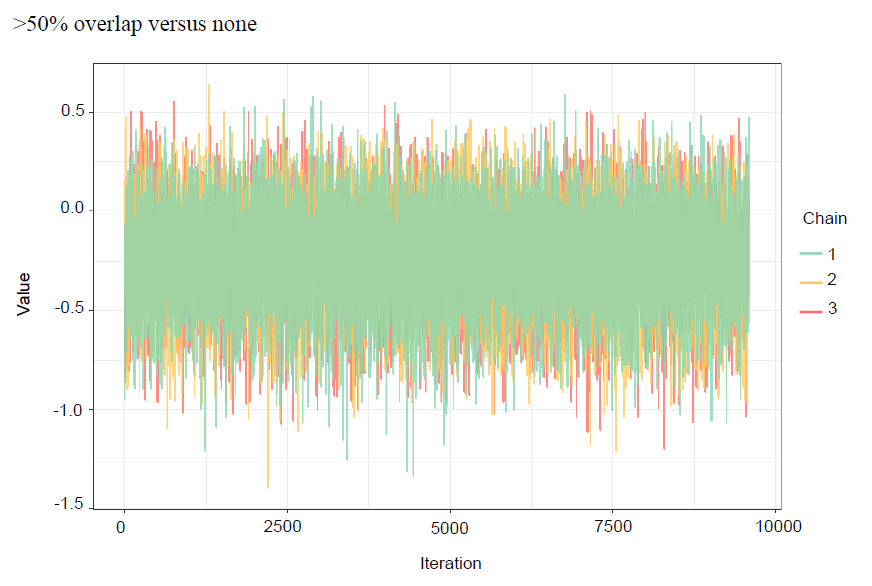


^†^ Adjusted for age, tumor stage, chemotherapy, radiotherapy, comorbidity, and simvastatin treatment.

## **Supplementary Figure S5**. Density plots for Bayesian joint model estimating the association between proportion overlap of all CYP2D6 inhibiting co-medications and breast cancer recurrence, among estrogen-negative positive premenopausal breast cancer patients.^†^


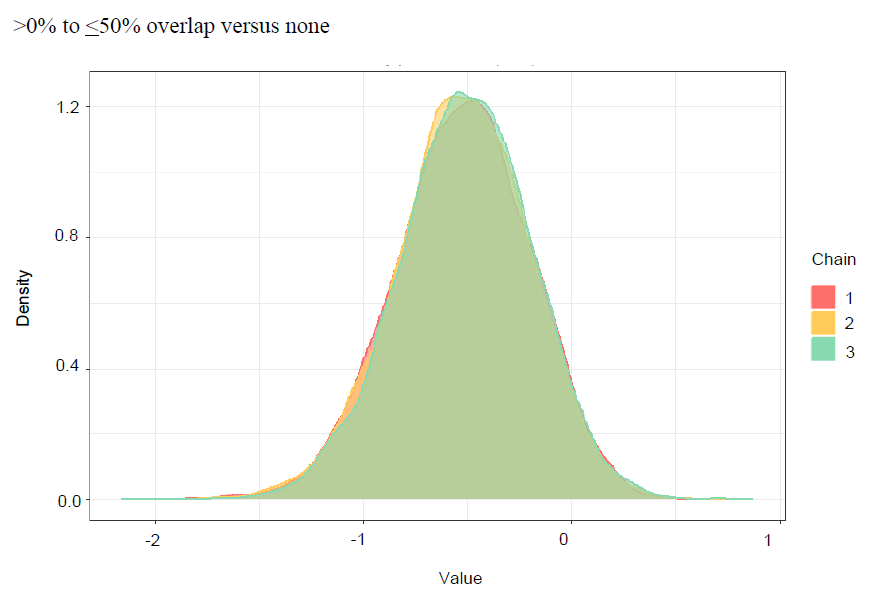


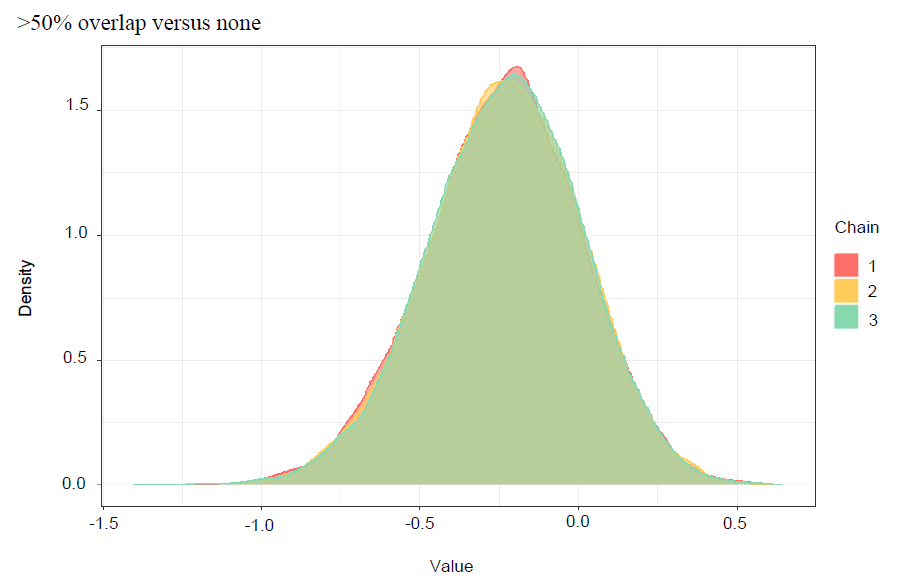


^†^ Adjusted for age, tumor stage, chemotherapy, radiotherapy, comorbidity, and simvastatin treatment.

**Supplementary Table S1**. Crude associations between proportion overlap of cytochrome P450-inhibiting co-medications and breast cancer recurrence among 4,493 estrogen receptor-positive (ER+) premenopausal women diagnosed with stage I, II, or III breast cancer between 2002-2011 in Denmark.


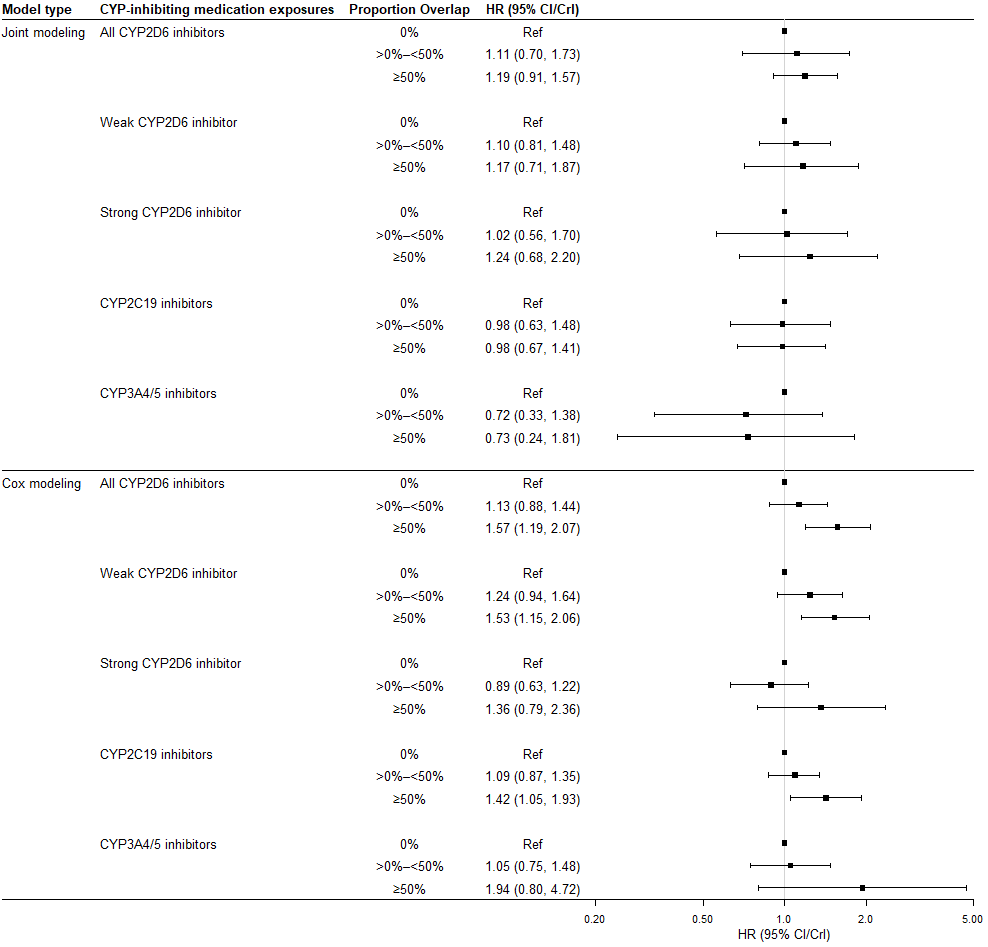


Abbreviations: ER, estrogen receptor; T, tamoxifen; CYP, cytochrome P450; HR, hazard ratio; CI, confidence interval; CrI, credible interval

**Supplementary Table S2**. Crude associations between proportion overlap of cytochrome P450-inhibiting co-medications and breast cancer recurrence among 1,204 estrogen receptor-negative (ER−) premenopausal women diagnosed with stage I, II, or III breast cancer between 2002-2011 in Denmark.


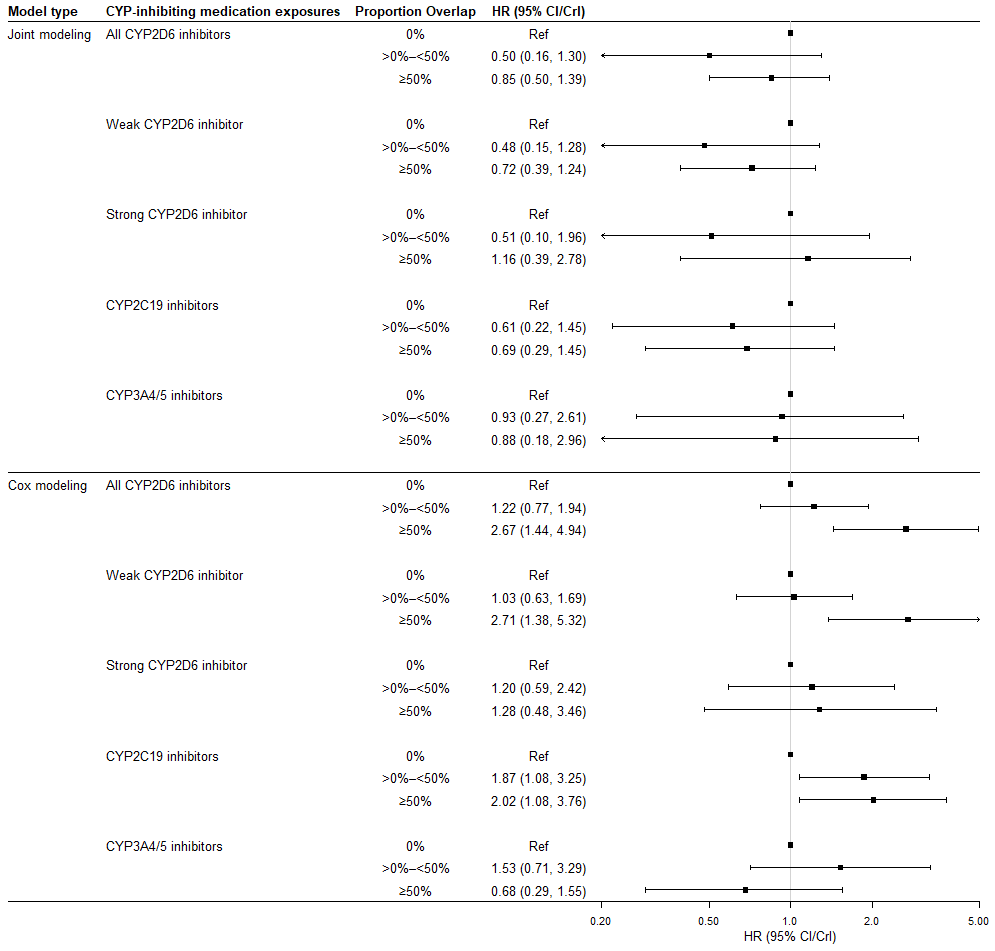


Abbreviations: ER, estrogen receptor; T, tamoxifen; CYP, cytochrome P450; HR, hazard ratio; CI, confidence interval; CrI, credible interval

**Supplementary Table S3**. Sensitivity analysis of associations between binary definition of cytochrome P450-inhibiting co-medications and breast cancer recurrence among 4,493 estrogen receptor-positive (ER+) premenopausal Danish women diagnosed with stage I, II, or III breast cancer between 2002–2011.

| **Modeling type** | **CYP-inhibiting**  **medication exposures** | **Crude hazard ratio** | **(95% CrI/CI)** | **Adjusted** ^†^  **hazard ratio** | **(95% CrI/CI)** |
| --- | --- | --- | --- | --- | --- |
| Joint modeling | Never any CYP inhibitor  Any CYP2D6 inhibitor  Weak CYP2D6 inhibitors  Strong CYP2D6 inhibitors  Any CYP2C19 inhibitor  Any CYP3A4/5 inhibitor | 1  1.11  1.10  1.10  0.84  0.46 | Ref  (0.71, 1.72)  (0.66, 1.75)  (0.42, 2.52)  (0.41, 1.61)  (0.07, 2.16) | 1  1.20  1.18  1.13  0.87  0.67 | Ref  (0.92, 1.55)  (0.88, 1.55)  (0.66, 1.83)  (0.57, 1.32)  (0.24, 1.69) |
| Cox modeling | Never any CYP inhibitor  Any CYP2D6 inhibitor  Weak CYP2D6 inhibitors  Strong CYP2D6 inhibitors  Any CYP2C19 inhibitor  Any CYP3A4/5 inhibitor | 1  1.00  1.02  0.65  1.02  1.65 | ref  (0.76, 1.32)  (0.76, 1.38)  (0.36, 1.20)  (0.74, 1.42)  (1.02, 2.66) | 1  0.98  1.00  0.62  0.99  1.82 | ref  (0.74, 1.30)  (0.74, 1.36)  (0.34, 1.13)  (0.71, 1.38)  (1.12, 2.96) |

^†^ Adjusted for age, tumor stage, chemotherapy, radiotherapy, comorbidity, and simvastatin treatment.

**Supplementary Table S4**. Sensitivity analysis of associations between binary definition of cytochrome P450-inhibiting co-medications and breast cancer recurrence among 1,204 estrogen receptor-negative (ER−) premenopausal Danish women diagnosed with stage I, II, or III breast cancer between 2002–2011.

| **Modeling type** | **CYP-inhibiting**  **medication exposures** | **Crude**  **hazard ratio** | **(95% CI)** | **Adjusted**^†^  **hazard ratio** | **(95% CI)** |
| --- | --- | --- | --- | --- | --- |
| Joint modeling | Never any CYP inhibitor  Any CYP2D6 inhibitor  Weak CYP2D6 inhibitors  Strong CYP2D6 inhibitors  Any CYP2C19 inhibitor  Any CYP3A4/5 inhibitor | 1  0.91  0.83  0.94  0.37  1.22 | Ref  (0.38, 1.98)  (0.33, 1.83)  (0.15, 3.89)  (0.07, 1.47)  (0.11, 8.30) | 1  0.94  0.86  0.98  0.43  1.19 | Ref  (0.57, 1.53)  (0.50, 1.44)  (0.35, 2.41)  (0.16, 1.05)  (0.22, 5.58) |
| Cox modeling | Never any CYP inhibitor  Any CYP2D6 inhibitor  Weak CYP2D6 inhibitors  Strong CYP2D6 inhibitors  Any CYP2C19 inhibitor  Any CYP3A4/5 inhibitor | 1  1.21  1.36  0.49  0.89  0.57 | ref  (0.85, 1.73)  (0.95, 1.96)  (0.18, 1.32)  (0.55, 1.45)  (0.21, 1.53) | 1  1.44  1.71  0.52  1.10  0.67 | ref  (0.99, 2.09)  (1.17, 2.50)  (0.19, 1.41)  (0.67, 1.82)  (0.25, 1.82) |

^†^Adjusted for age, tumor stage, chemotherapy, radiotherapy, comorbidity, and simvastatin treatment.

## **Supplementary Table S5**. Associations between proportion overlap of cytochrome P450-inhibiting co-medications and breast cancer recurrence, according to estrogen receptor expression and tamoxifen treatment status. Estimated with Bayesian joint models. Sensitivity analysis with person-time at risk starting six months after diagnosis.


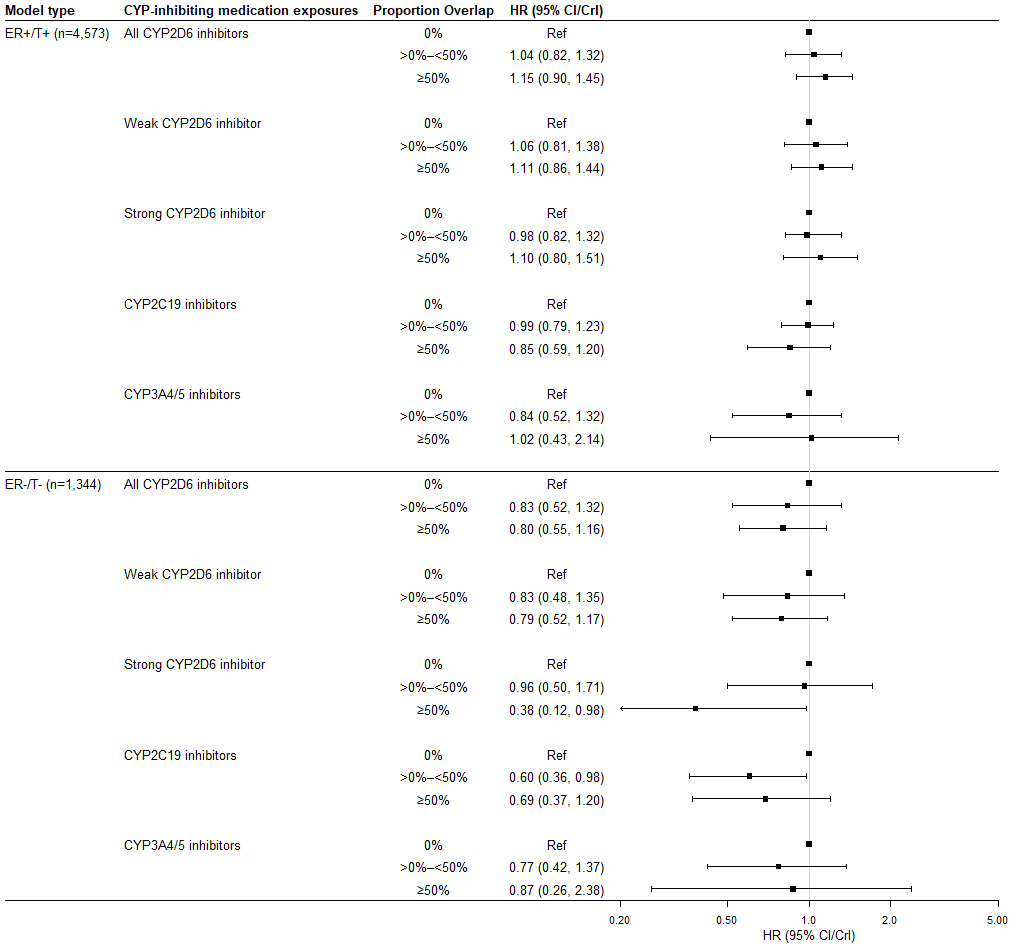


Abbreviations: ER, estrogen receptor; T, tamoxifen; CYP, cytochrome P450; HR, hazard ratio; CI, confidence interval; CrI, credible interval

**Supplementary Table S6**. Associations between proportion overlap of CYP2C19 and CYP3A4/5 inhibiting co-medications and breast cancer recurrence, according to estrogen receptor expression and tamoxifen treatment status. Estimated with Bayesian joint models. Sensitivity analysis with 30-day supply assumption.

| **ER / tamoxifen status** | **CYP-inhibiting**  **medication exposures** | **Proportion overlap** | **Adjusted**^†^  **hazard ratio** | **(95% CI)** |
| --- | --- | --- | --- | --- |
| ER+/TAM+ | CYP2C19 inhibitors  Any CYP3A4/5 inhibitor | 0%  >0%−<50%  ≥50%  0%  >0%−<50%  ≥50% | 1  0.99  0.87  1  0.92  . | ref  (0.80, 1.21)  (0.37, 1.78)  ref  (0.63, 1.31  . |
| ER-/TAM- | CYP2C19 inhibitors  Any CYP3A4/5 inhibitor | 0%  >0%−<50%  ≥50%  0%  >0%−<50%  ≥50% | 1  0.56  .  1  0.82  . | ref  (0.32, 0.88)  .  ref  (0.38, 1.57)  . |

^†^Adjusted for age, tumor stage, chemotherapy, radiotherapy, comorbidity, and simvastatin treatment.

**References**

1. Wang W, Yan J. Shape-Restricted Regression Splines with R Package splines2. | Journal of Data Science | EBSCOhost [Internet]. 2021 [cited 2024 May 31];19(3):498. Available from: https://openurl.ebsco.com/contentitem/doi:10.6339%2F21-JDS1020?sid=ebsco:plink:crawler&id=ebsco:doi:10.6339%2F21-JDS1020

2. Alsefri M, Sudell M, García-Fiñana M, Kolamunnage-Dona R. Bayesian joint modelling of longitudinal and time to event data: a methodological review. BMC Med Res Methodol 2020;20:94.

3. Asar Ö, Ritchie J, Kalra PA, Diggle PJ. Joint modelling of repeated measurement and time-to-event data: an introductory tutorial. Int J Epidemiol 2015;44(1):334–44.

4. Hatfield LA, Boye ME, Carlin BP. Joint modeling of multiple longitudinal patient-reported outcomes and survival. J Biopharm Stat 2011;21(5):971–91.
